# Replay of Learned Neural Firing Sequences during Rest in Human Motor Cortex

**DOI:** 10.1016/j.celrep.2020.107581

**Published:** 2020-05-05

**Authors:** Jean-Baptiste Eichenlaub, Beata Jarosiewicz, Jad Saab, Brian Franco, Jessica Kelemen, Eric Halgren, Leigh R. Hochberg, Sydney S. Cash

**Affiliations:** 1 Center for Neurotechnology and Neurorecovery, Department of Neurology, Massachusetts General Hospital, Boston, MA, USA; 2 Department of Neurology, Harvard Medical School, Boston, MA, USA; 3 Department of Neuroscience, Brown University, Providence, RI, USA; 4 Carney Institute for Brain Science, Brown University, Providence, RI, USA; 5 VA RR&D Center for Neurorestoration and Neurotechnology, Rehabilitation R&D Service, Department of VA Medical Center, Providence, RI, USA; 6 School of Engineering, Brown University, Providence, RI, USA; 7 Departments of Radiology and Neuroscience, Kavli Institute for Brain and Mind, University of California, San Diego, CA, USA; 8 These authors contributed equally; 9 Present address: NeuroPace, Inc., Mountain View, CA, USA; 10 Lead Contact

## Abstract

The offline “replay” of neural firing patterns underlying waking experience, previously observed in non-human animals, is thought to be a mechanism for memory consolidation. Here, we test for replay in the human brain by recording spiking activity from the motor cortex of two participants who had intracortical microelectrode arrays placed chronically as part of a brain-computer interface pilot clinical trial. Participants took a nap before and after playing a neurally controlled sequence-copying game that consists of many repetitions of one “repeated” sequence sparsely interleaved with varying “control” sequences. Both participants performed repeated sequences more accurately than control sequences, consistent with learning. We compare the firing rate patterns that caused the cursor movements when performing each sequence to firing rate patterns throughout both rest periods. Correlations with repeated sequences increase more from pre- to post-task rest than do correlations with control sequences, providing direct evidence of learning-related replay in the human brain.

## In Brief

Eichenlaub et al. show that in the motor cortex of brain-computer interface trial participants, the firing rate patterns corresponding to a previously learned motor sequence are replayed during rest. These findings provide direct evidence of memory replay in the human brain.

## Graphical Abstract

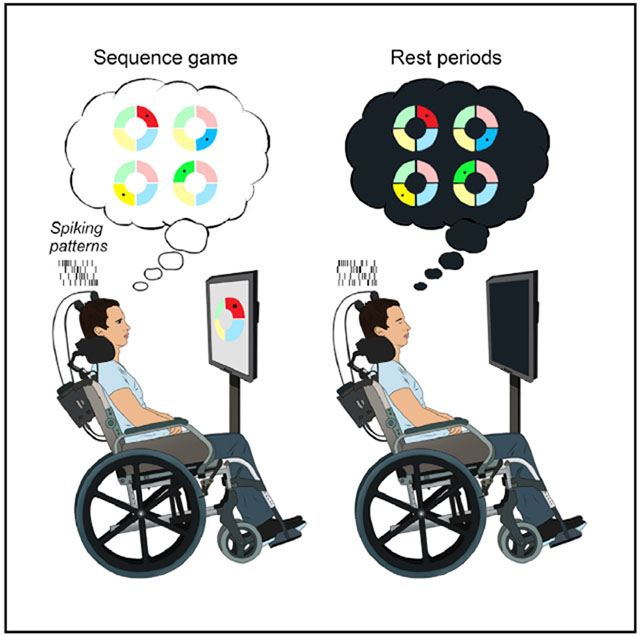

## INTRODUCTION

There is strong convergent evidence from behavioral, cognitive, lesion, and computational studies that following the initial encoding of new memories, offline mechanisms–yet to be fully elucidated–are involved in their long-term consolidation (Abel et al., 2013; Atherton et al., 2015). The offline “replay” of neural firing sequences that occurred during learning has been proposed as a potential mechanism of memory consolidation and was initially described in rodents whose hippocampal “place cells” were found to fire in the same order during rest as they had while the animals ran along a recently traversed path (Pavlides and Winson, 1989; Skaggs and McNaughton, 1996; Wilson and McNaughton, 1994). Following up on this seminal work, memory replay has been shown in different cortical and subcortical areas in non-human animals, including in the visual cortex (Ji and Wilson, 2007), ventral striatum (Lansink et al., 2009), medial prefrontal cortex (Euston et al., 2007), parietal cortex (Wilber et al., 2017), and motor cortex (Gulati et al., 2014; Ramanathan et al., 2015); and during wakefulness (Csicsvari et al., 2007; Davidson et al., 2009; Diba and Buzsáki, 2007; Foster and Wilson, 2006; Karlsson and Frank, 2009), non-rapid eye movement (NREM) sleep (Gulati et al., 2014; Ji and Wilson, 2007; Lansink et al., 2009; Lee and Wilson, 2002; Ramanathan et al., 2015; Ribeiro et al., 2004; Wilber et al., 2017), and REM sleep (Louie and Wilson, 2001).

In humans, non-invasive functional neuroimaging studies have provided indirect evidence for replay. First, by presenting associated odor cues (i.e., odor presented as context during prior learning) during NREM (slow-wave) sleep, a set of studies reported that odor re-exposure was associated with hippocampal activation during sleep (Diekelmann et al., 2011; Rasch et al., 2007). Second, several studies reported the reactivation of learning-related cerebral activity during offline periods, such as a post-learning increase in hippocampal activation during slow-wave sleep (Peigneux et al., 2004) or an increase in learning-induced magnetoencephalography (MEG) synchronization during sleep (Piantoni et al., 2015). Reactivation has also been observed in motor areas following motor learning, including an increase in premotor cortex activity during REM sleep (Maquet et al., 2000). Third, using pattern analysis applied to fMRI signals, recent studies reported the reactivation of item-specific “engram patterns” (i.e., voxel activity patterns specific to individual learning experiences) (Deuker et al., 2013; Schapiro et al., 2018; Staresina et al., 2013; Tambini and Davachi, 2013; Zhang et al., 2017).

More recently, two studies took advantage of intracranial macro-electrode recordings (iEEGs) taken during presurgical epilepsy monitoring to reveal the re-occurrence of spatio-temporal gamma activity patterns from waking during subsequent rest and sleep (Jiang et al., 2017; Zhang et al., 2018). Jiang et al. (2017) identified recurrent spatiotemporal sequences of gamma activity peaks across widespread cortical regions (“motifs”) during waking periods and found more closely matching sequences during subsequent sleep than preceding sleep periods. Furthermore, motifs occurring during the performance of a cognitive task were more likely to have more matches in subsequent sleep (Jiang et al., 2017). Similarly, Zhang et al. (2018) identified item-specific gamma activation patterns during a visual memory task performed before and after an afternoon nap, and they reported a higher level of reactivation of the before-nap items’ gamma patterns than the after-nap items’ gamma patterns. This reactivation was observed during both waking and sleep, and for both remembered and forgotten items, with the depth of encoding reflected in qualitative differences in ripple-triggered reactivation in NREM (Zhang et al., 2018).

Both sets of findings are consistent with offline memory replay in the human brain, but non-invasive imaging and iEEG do not have the spatial resolution to directly test for replay at the level of neural firing rate patterns. Here, we recorded from large ensembles of single units in motor cortex of research participants with intracortical microelectrode arrays to seek direct evidence of offline replay of specific learning-related neural firing rate sequences. Two participants with tetraplegia (T9 and T10) each had two 96-channel silicon microelectrode arrays (BlackRock Microsystems, Salt Lake City, Utah, USA) placed neurosurgically as part of the BrainGate2 pilot clinical trial (https://www.clinicaltrials.gov/ct2/show/NCT00912041). This clinical trial is part of the ongoing effort to develop brain-computer interfaces (BCIs) that allow people with tetraplegia to control computer cursors, tablet computers, robotic arms, and other devices with their neural activity (Aflalo et al., 2015; Ajiboye et al., 2017; Bouton et al., 2016; Collinger et al., 2013; Gilja et al., 2015; Hochberg et al., 2006, 2012; Jarosiewicz et al., 2015; Nuyujukian et al., 2018; Truccolo et al., 2008). Intermixed with sessions dedicated to other neuroprosthetics research, each participant performed five research sessions testing for memory replay. The BCI paradigm confers a unique advantage for studying memory replay because the recorded neural activity patterns that are tested for replay are known to have been directly and causally responsible for the performance of the learned motor sequence.

## RESULTS AND DISCUSSION

### Repeated Brain-Controlled Sequences Were Learned over and above Control Sequences

**Figure 1.**
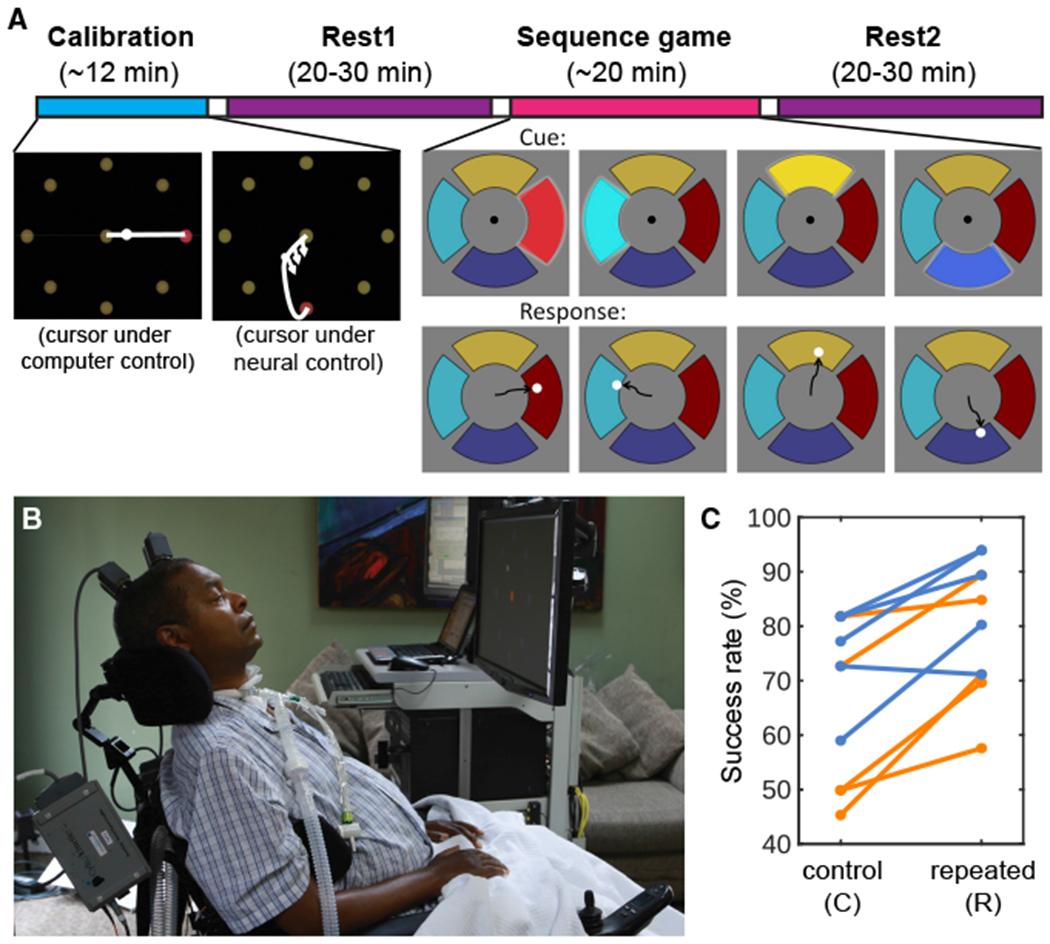
Research Session Setup (A) Task and timeline. In each session, a decoder was initialized and calibrated using an open-loop and then closed-loop center-out task (see Method Details; calibration inset modified from Jarosiewicz et al., 2015). The participant was then invited to relax with his eyes closed, and nap if desired, during a pre-task rest period (Rest1). Following Rest1, the participant performed the 4-target sequence game, consisting of 66 presentations of the repeated sequence (in this example, red-teal-yellow-blue), interspersed with 22 control sequences. Following the game, the participant was invited to relax with his eyes closed again (Rest2). (B) Participant T9 at his home during a research session. (C) Performance in the game, divided into control and repeated sequences. Each line represents one session from one participant (orange = T9 sessions; cornflower blue = T10 sessions). The success rate (% of sequences correctly completed) was significantly higher for the repeated than the control sequences (Wilcoxon signed-rank test, n = 10, p = 0.0039), suggesting preferential learning of the repeated sequences.

**Figure 2.**
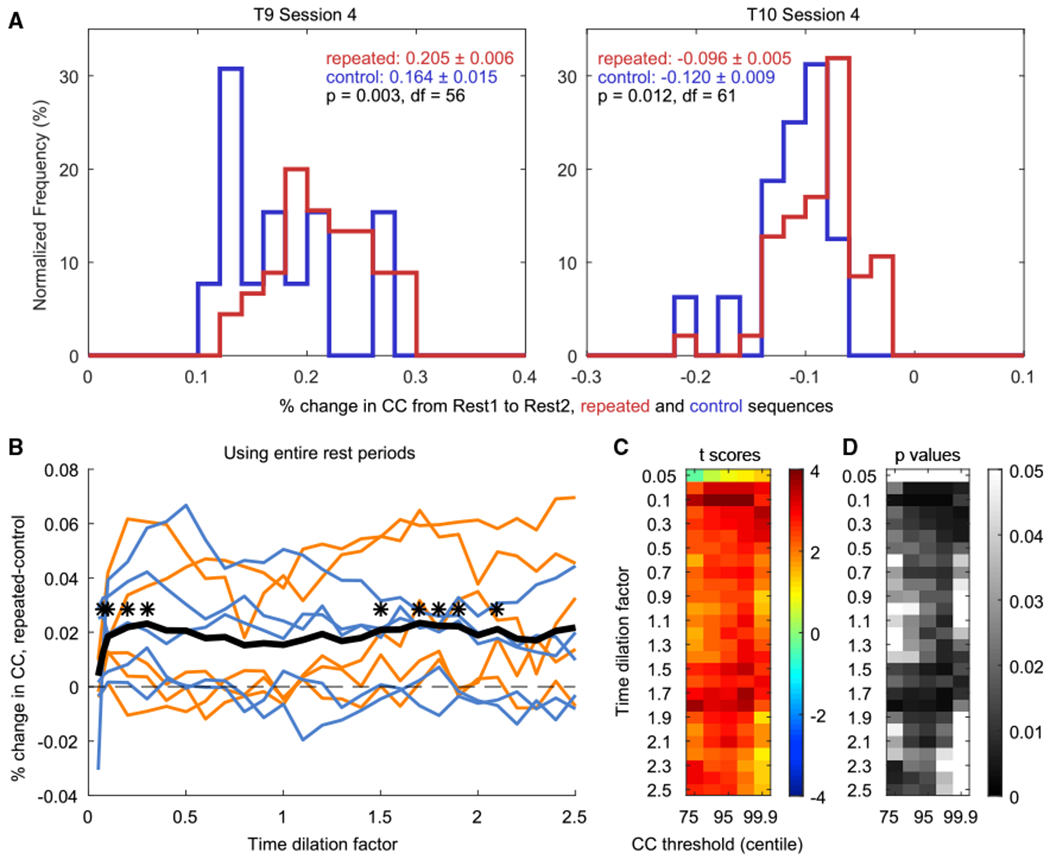
Replay of Firing Rate Patterns in Motor Cortex (A) Examples of distributions of RIs. One value in each distribution is the RI for one trial (the *%* change in mean peak CC values from Rest1 to Rest2 when using that trial as the template). The red distributions were obtained using the *repeated* sequence trials as templates, and the blue distributions were obtained using the *control* trials. Neural signal nonstationarities can cause spurious drifts in CC values; thus, replay was measured as the *difference* between the repeated and the control trials’ RIs. In both example sessions (one from each participant), the repeated sequence trials (mean ± SEM in red) showed significantly higher (i.e., more positive) RIs than the control sequence trials (in blue; two-sample one-tailed t test). (B) Difference in mean RI (repeated – control) for each session at all time dilation factors (using a CC threshold at the 95th centile). Sessions with T9 are shown in orange and those with T10 in cornflower blue. The mean across sessions is shown with a thick black line. Asterisks denote time dilation factors at which the *repeated*-trial mean RIs were significantly higher than the *control*-trial mean RIs across sessions (paired one-tailed t test, n = 10; *p < 0.01). (C and D) The t scores (C) and corresponding p values (D) resulting from testing, for each time dilation factor and centile threshold, whether the *repeated*-trial mean RIs were higher than the *control*-trial mean RIs across sessions (paired one-tailed t test, n = 10). Note that the middle columns of (C) and (D) correspond to the data shown in (B).

Each replay session (Figure 1) began with a standard “center-out-back” decoder calibration task in which targets were presented one by one on a computer screen. The participant was asked to imagine moving their hand to control the cursor as it moved to the targets automatically and then to move the cursor in real time using their neural activity decoded using a Kalman filter (Figures 1A, 2 [leftmost screen shots], and 1B; see also Method Details). The 40 neural features (either threshold-detected action potentials or the total signal power in the spike band, a continuous proxy for firing rates; Jarosiewicz et al., 2015) that had the highest tuning to intended movement direction (Malik et al., 2015) during the calibration task were used for controlling the cursor in real time during the sequence game and to test for replay during rest. We will call both sets of neural features “spike rates” for simplicity.

On each trial of the sequence game, a specific sequence of four radially displaced wedge-shaped targets was presented, and the participant was asked to move a cursor under neural control from the center of the screen to the same sequence of four targets as quickly and accurately as possible (Figure 1A; see also Videos S1 and S2). In a given research session, the same “repeated” sequence was presented in 66 trials, pseudo-randomly interleaved with 22 “control” sequences (11 unique sequences, each repeated twice) that did not include any of the same target transitions as the repeated sequence. A different repeated sequence was used in each session for each participant. A 20–30-min rest period took place before (Rest1) and after (Rest2) the sequence game, during which neural signals were recorded as the participant was invited to close his eyes, relax, and nap if desired.

To test for learning, the success rate (the percentage of 4-target sequences that were correctly acquired) and time to complete each sequence were compared between the repeated and control sequences. When successful, there was no difference in the amount of time it took to complete the repeated versus control sequences (mean ± SEM, repeated sequences: 4.50 ± 0.21 s; control sequences: 4.47 ± 0.25 s; Figure S1). However, the success rate was higher for the repeated (mean ± SEM: 80.2% ± 3.9%) than for the control sequences (67.3% ± 4.6%; Wilcoxon signed-rank test, n = 10, p = 0.0039; Figure 1C), providing evidence for learning of the repeated sequence beyond the general improvement at the game expected from practice.

### Sequenced Replay of Single Targets

To test for replay of these learned sequences in motor cortex, the firing rates of the same 40 features that had been used for decoding were examined during the rest periods. On average (mean ± SD), the total amount of time spent in Rest1 and Rest2, respectively, was 23.51 ± 2.41 min and 26.15 ± 2.89 min. First, each channel’s features were adaptively *Z* scored (see Method Details) to reduce the effects of any nonstationarities in feature means and SDs (Jarosiewicz et al., 2016; Perge et al., 2013) that might otherwise have spuriously affected correlations across time. Then, the average neural activity pattern corresponding to each *single target* was extracted from the repeated sequence trials in each session; i.e., all trajectories to a given target were temporally resampled to their mean timescale, and their corresponding neural patterns were averaged together.

The average of the neural activity pattern for each target was used as an individual spatiotemporal “template” that was compared to each timestep of Rest1 and Rest2 using normalized 2D cross-correlation (described in Tatsuno et al., 2006; see also Method Details). Local peaks in the correlation coefficient (CC) time series above the 95th centile (candidate replay events) were identified, and non-maximum suppression (see, e.g., Felzenszwalb et al., 2010; Rothe et al., 2015) with a window size equal to the template duration was used to allow, at most, one local peak per template length (see Method Details). The single-target CC peaks were then interleaved into a single time series, with each peak labeled with its target ID (1, 2, 3, or 4). Each consecutive set of four targets (irrespective of their temporal spacing) was identified as either matching the order of targets in the repeated sequence (hits) or not (misses).

The number of hits as a proportion of the total number of 4-target sequences (hits + misses) in each rest block was accumulated across all sessions for Rest1 and separately for Rest 2. Across sessions, the proportion of 4-target hits corresponding to the repeated sequence was significantly higher in Rest2 (75/11,033, or 0.68%) than in Rest1 (34/8703, or 0.39%; Chi-square proportion test, Chi^2^(1) = 7.40; p = 0.0065). In contrast, combining all *control* sequences across all sessions, the proportion of control sequence hits did not differ between Rest1 (606/95,733, or 0.63%) and Rest2 (741/121,363, or 0.61%; Chi-square proportion test, Chi^2^(1) = 0.437; p = 0.51; n.s.). These results demonstrate the presence of temporally sequenced replay that is specific to the *repeated* sequences.

### Replay of Full 4-Target Neural Activity Patterns

The sequenced single-target replay analysis described above is not sensitive to the timing of the individual-target matches; the only relevant factor is the relative *sequence* of single-target matches. In fact, in the above analysis, the durations of the 4-individual-target replay events have a lower limit determined by the window size of the non-max suppression algorithm, which was set to the mean length of each target’s trajectory during the task. To check for the timing of replay of the neural activity corresponding to the entire 4-target sequence, we created templates from the neural sequences obtained during the task and compared these templates to the rest blocks using cross-correlation.

Typically, the template-matching approach involves averaging the neural activity across trials to create a single template (see, e.g., Euston et al., 2007; Wilber et al., 2017), as we did in the section above for the individual-target sequenced replay analysis. However, approximating the learning-related firing rate patterns with a single time-aligned average (1) can introduce warping and artifact, (2) discards information about trial-to-trial variability, and (3) is subject to spurious results attributable to, for example, nonstationarities in signal statistics (see, e.g., Tatsuno et al., 2006). Human intracortical micro-recordings in particular often have prominent neural signal nonstationarities (see, e.g., Jarosiewicz et al., 2016; Perge et al., 2013), which could potentially artificially inflate (or deflate) the measure of replay, even with adaptive *Z* scoring. For example, if electrical contact with one or more neurons is lost (or gained) between Rest1 and the sequence task due to local brain micromovement relative to the electrode, this could deflate the correlation between Rest1 and the task, thus artificially inflating the relative correlation between the task and Rest2.

In the template-matching approach, shuffle tests are commonly used to create control templates. However, because no shuffled scenario represents veridical neural activity-task pairings, no single shuffle test is a perfect control on its own. Thus, multiple different shuffle tests are often performed, and the most conservative of them is sometimes selected for reporting statistical significance (see, e.g., Louie and Wilson, 2001). However, even the most conservative shuffle test retains the possibility of being dependent on an incorrect assumption about the integrity of the remaining, unshuffled statistics.

To avoid these shortcomings, we introduce a more conservative approach to testing for replay by using *each trial’s* neural activity as a separate template and comparing the distribution of replay results using the templates corresponding to the *repeated* (learned) sequences versus the *control* sequences. This approach bypasses the need for shuffle tests, because the control sequences reflect *veridical* neural activity obtained under conditions as close as possible to, and temporally interleaved with, the learned sequences: the control neural patterns arise from movements to the same set of four targets but in a different (unlearned) order. Because they were temporally interleaved throughout the task, nonstationarity cannot spuriously inflate or deflate correlations for the learned sequence without similarly affecting the control sequences. Finally, using each trial as a separate template preserves veridical neural activity and allows trial-to-trial variability to be reflected in the widths of the distributions of the replay measures, allowing for straightforward statistical comparisons in the replay of the repeated versus control sequences.

To perform this analysis, after adaptive *Z* scoring, each 4-target trial was used as a separate spatiotemporal template that was compared to each timestep of Rest1 and Rest2 using (as before) normalized 2D cross-correlation. For each template, CC peaks above the 95th centile (candidate replay events) were extracted, and using non-maximum suppression (as above) ensured a maximum of 1 CC peak per template length (see Method Details; Figure S2). For each template, we then computed the mean of CC peak values in Rest1 (m_1_) and in Rest2 (m_2_), respectively, and then defined the percentage change in the mean CC peak values from Rest1 to Rest2 as the replay index (RI) = (m_2_ – m_1_) / m_1_. This process was repeated for all trials, resulting in one distribution of RIs for the repeated sequence trials and one for the control trials (Figure 2A). For each session, we then tested whether the RIs were higher for the repeated sequences than the control sequences using a two-sample one-tailed t test (Figures 2A and S3).

To test for replay across all 10 sessions from the two participants, we computed the mean RI of the *repeated* sequence trials and the mean RI of the *control* sequence trials for each session and tested whether the *repeated*-trial mean RIs were significantly higher than the *control*-trial mean RIs across sessions (Figures 2B–2D). In rodents, replay (or consistent bias toward waking firing patterns) has been shown to occur at timescales near those of the original neural patterns recorded during learning (Louie and Wilson, 2001; Ribeiro et al., 2004) and also at 5–20× faster timescales (Csicsvari et al., 2007; Davidson et al., 2009; Diba and Buzsáki, 2007; Euston et al., 2007; Foster and Wilson, 2006; Ji and Wilson, 2007; Karlsson and Frank, 2009; Lansink et al., 2009; Lee and Wilson, 2002; Wilber et al., 2017). Thus, to check for replay events at different timescales, we resampled each template to 0.05×– 2.5× the actual duration of each trial and recomputed the RI for each template at each timescale. We then proceeded as before, testing across sessions whether the mean RIs for the *repeated* sequence trials at each timescale were larger than the mean RIs for the *control* sequence trials at that same timescale. Consistent with the rodent studies, we found significant replay (p < 0.01) peaking at timescales of roughly 0.1× the actual duration of the trials (i.e., ~10× faster than real time) and ~1.5–2× slower (Figure 2B).

### Controlling for Centile Threshold and Multiple Comparisons

**Figure 3.**
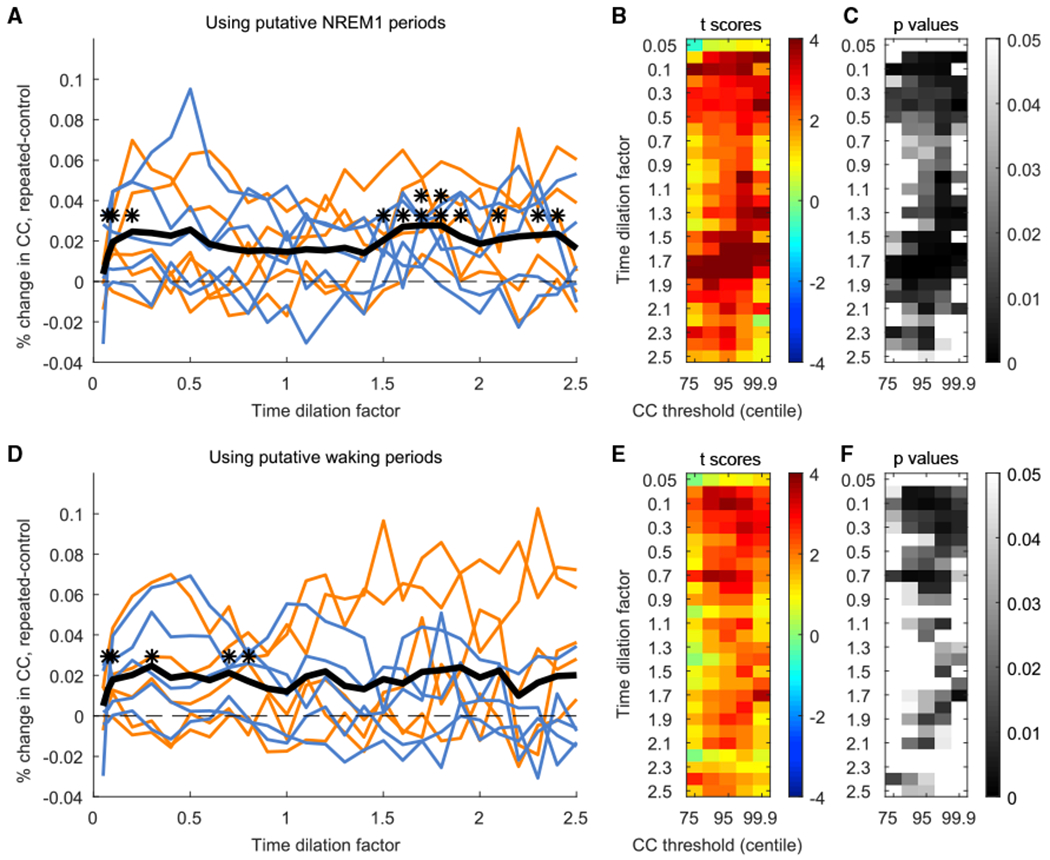
Replay Results across Sessions, with Rest Periods Subdivided into Putative NREM1 and Putative Waking States (A–C) Analagous to Figures 2B–2D, showing results for putative NREM1. (D–F) Analagous to Figures 2B–2D, showing results for putative waking. *p < 0.01; **p < 0.001.

To verify that these results were not sensitive to the particular CC centile threshold we used (the 95th centile) to define CC peaks, we repeated the same analysis using centile thresholds of 75, 90, 99, and 99.9. Across sessions, we found that significant replay generalized to some degree across centile thresholds (Figures 2C and 2D). Higher thresholds reduce the number of peaks being compared, thus reducing statistical power, whereas lower thresholds add noise; this sweep confirmed that the 95th centile (used in Figures 2B, 3A, and 3D) provides a reasonable tradeoff between statistical power and signal-to-noise ratio. Figure S3 shows the results of comparing the control versus repeated sequence trials for all combinations of centile thresholds and timescales for each session individually.

Although multiple statistical comparisons were performed to produce Figures 2B–2D, they were not fully independent because neighboring comparisons came from overlapping data (e.g., templates stretched to neighboring time dilations are nearly identical to each other; similarly, the set of peaks above any particular centile threshold includes all of the peaks above higher centile thresholds). Thus, a standard multiple-comparison correction does not readily apply to this analysis. Instead, to confirm that these results were not simply a spurious consequence of multiple comparisons, we also performed the same number of *swapped* t tests, checking whether we observed significantly higher RIs for the *control* sequences than for the *repeated* sequences across sessions. We found no significant differences at any timescale or centile threshold using the same analysis with the *control* and *repeated* RIs swapped, demonstrating that the significant replay was not simply a consequence of the large number of comparisons performed (Figure S4).

### Replay of Full 4-Target Neural Activity Patterns during Both Putative Waking and NREM1 States

The above analyses (Figure 2) were performed using the entire rest periods, irrespective of variation over time in sleep/waking states. Although memory replay was initially associated with sleep, mounting evidence suggests a complementary role for waking replay in memory consolidation (Carr et al., 2011). It has been proposed that early waking replay could serve to initially stabilize memories, and later sleep replay could establish long-term memories by consolidating the underlying connections among multiple cortical regions (Squire et al., 2015). Evidence of replay in humans during waking would be of great importance in the memory consolidation theory: because the human sleep-wake cycle follows a roughly 24-h rhythm, many hours can elapse between encoding and sleep. To test whether the characteristics of human replay are affected by sleep/waking state, we subdivided the rest blocks into putative sleep and waking periods and compared the replay of the repeated sequences to the control sequences separately for each period.

Short, daytime naps (<30 min) are largely composed of NREM sleep, mainly stages N1 and N2 (Hayashi et al., 2005). In addition, visual examination of the local field potentials (LFPs) collected from the implanted arrays during the rest blocks confirmed the occurrence of periods of NREM1 sleep, marked by increases in theta power (4–7 Hz). Accordingly, we quantified the instantaneous theta amplitude in the LFP (see Method Details; Figure S5) and defined “putative NREM1” periods as those with high theta amplitude and “putative waking” periods as those with low theta amplitude. The amount of time each participant spent in putative NREM1 and putative waking in each session is listed in Table S2. Consistent with animal studies, the repeated sequence trials showed significantly higher replay than did the control sequence trials during both putative NREM1 and waking periods (Figure 3). Our results, together with previous findings, are consistent with replay during waking supporting a shorter-delay consolidation for memories acquired earlier in the day.

In rodents, replay events often co-occur with hippocampal sharp-wave ripples (SWRs), a transient hippocampal activity that is observed in both wakefulness and sleep (for a review, see Buzsáki, 2015). Although humans also exhibit hippocampal SWRs, their density is low during waking and NREM1 (Jiang et al., 2019a). They occur predominantly in deeper NREM sleep (N2 and N3), often in conjunction with recurrent spatiotemporal sequences of gamma activity peaks (Jiang et al., 2017), just before cortical spindles and upstates, and just after theta bursts and downstates (Jiang et al., 2019a, 2019b). Because SWRs and their associated cortical events have been linked to replay in rodents, replay in humans may also be stronger in deeper sleep than we observed here in putative NREM1 and waking states.

Although both putative NREM1 and waking periods exhibited temporally compressed replay peaking at timescales of roughly 0.1× the actual duration of the trials, putative NREM1 periods additionally exhibited temporally dilated replay (peaking at timescales of 1.5–2×), consistent with timescales previously reported during REM sleep in rats (Louie and Wilson, 2001). Additionally, the timescales of replay observed in the current study are broader than typically observed in rodents, in whom replay tends to be observed in a restricted set of timescales during SWRs. This difference could be a reflection of circuit interactions (for a review, see Buzsáki, 2015) that might differ between the rat and human cortex. The temporal features of the replay of firing rate patterns in deeper NREM (NREM2 and NREM3), which contain a higher density of SWRs and therefore might be more analogous to slow-wave sleep in the rodent, remain to be characterized in human studies.

In summary, neural firing rate patterns corresponding to a previously learned BCI-controlled motor sequence are replayed in the human cortex during rest. Replay occurs at multiple timescales related to the ongoing physiological state and includes the specific units whose activity was causally responsible for performing the motor sequences. Future studies are needed to address many outstanding questions. For example, at what timescales does replay occur in humans during overnight sleep, including deeper NREM and REM sleep? Is there temporal coupling between human replay events and cardinal sleep oscillations, such as neocortical slow oscillations and hippocampal SWRs? Does the salience or relevance of the encoded event, or the person’s motivational or emotional context during encoding, modulate the strength of replay? Even though the use of human microscale neural recording systems is rare and, at present, limited to investigational use (Cash and Hochberg, 2015), exploring the attributes of human memory replay and its relationship to behavioral and cognitive aspects of learning and memory is likely to help us better understand how human beings learn from experience.

## STAR★METHODS

### RESOURCE AVAILABILITY

#### Lead Contact

Requests for resources should be directed to the Lead Contact, Jean-Baptiste Eichenlaub (jb.eichenlaub@gmail.com).

#### Materials Availability

This study did not generate new unique reagents.

#### Data and Code Availability

All reasonable requests for collaboration involving materials used in the research will be fulfilled provided that a written agreement is executed in advance between MGH and the requester (and his or her affiliated institution).

### EXPERIMENTAL MODEL AND SUBJECT DETAILS

Permission for this study was granted by the U.S. Food and Drug Administration (Investigational Device Exemption #G090003) and the Institutional Review Boards of Partners Healthcare/Massachusetts General Hospital, Providence VA Medical Center, and Brown University. The two participants (identified as T9 and T10, Table S1) had two 96-channel intracortical silicon microelectrode arrays (Blackrock Microsystems, Salt Lake City, Utah, US) placed neurosurgically as part of the BrainGate2 pilot clinical trial (https://www.clinicaltrials.gov/ct2/show/NCT00912041). Both participants gave informed consent to the study and publications resulting from the research, including consent to publish photographs and audiovisual recordings of them.

Both participants were proficient at controlling motor imagery-based brain computer interfaces (BCI), and took part in 1-3 sessions (3-4 hours each) per week, of which the current study was one of several concurrent studies.

Participant T9 was a right-handed man, 52 years old at the start of the study, with ALS with a functional scale rating (ALSFRS-R) of 8. He retained speech, breathed with the assistance of a mechanical ventilator, and had very limited hand movement. He received two 1.5 mm 96-channel intracortical silicon microelectrode arrays (Blackrock Microsystems), as described previously (Hochberg et al., 2006), in the dominant hand/arm area of motor cortex (Yousry et al., 1997) approximately 11 months before the start of his participation in this research.

Participant T10 is a right-handed man, 34 years old at the start of the study, with tetraplegia due to cervical spinal cord injury. He retains speech, breathes with the assistance of a mechanical ventilator, and has no limb or hand movement. He received two 1.5 mm 96-channel intracortical silicon microelectrode arrays (Blackrock Microsystems); one in the hand/arm area of motor cortex and one in the middle frontal gyrus, approximately 3 months before the start of his participation in this research.

### METHOD DETAILS

#### Signal acquisition

Neural activity detected by the 96 recording channels of each microelectrode array was transmitted via a cable attached to a percutaneous connector during each recording session. Signals were analog filtered (4th order Butterworth with corners at 0.3 Hz and 7.5 kHz) and digitized at 30 ksps by two 128-channel NeuroPort Neural Signal Processors (Blackrock Microsystems, Salt Lake City, Utah, US). The signals from both systems were fed to custom software written in Simulink (The MathWorks, Inc.) for saving and for further processing and decoding.

#### Signal pre-processing

For pre-processing, signals were downsampled to 15 kHz and then common-average referenced (Ludwig et al., 2009) to reduce any electrical artifacts common to all channels. Namely, the mean signal across the 60 channels with the lowest noise distribution on each array, identified from a single 30 s “reference” recording at the start of the first session, was computed and subtracted in real time from all channels’ signals on that array. In 20 ms segments, signals were buffered for 4 ms to avoid edge effects, and then non-causally band-pass filtered for action potentials (spikes) using a 4th order Butterworth filter with corners at 250 and 5000 Hz (Masse et al., 2014).

#### Neural feature extraction

The total spectral power in the spike frequency band (250-5000Hz) on each channel was used as one set of neural features (called “spike power”), and can be thought of as a continuous estimate of multi-unit spike rate. To isolate large-amplitude spikes from lower-amplitude spikes and background noise, the spike-band signal was also compared to an amplitude threshold of −3.5 x the standard deviation of the filtered signal on each channel (Chestek et al., 2011; Christie et al., 2015; Fraser et al., 2009; Masse et al., 2014) and the rate of these “threshold-crossing” events, also called “multi-unit spiking activity,” was used as a 2nd set of neural features. These neural features, both of which we will call “spike rates” for simplicity, were used as separate candidate input features for decoding (see decoder calibration sections below for details). In participant T9, both sets of features were used. In participant T10, only spike power was used.

#### Kalman filter

Cursor movement intention was decoded from neural features using a steady-state Kalman filter, as described previously (Jarosiewicz et al., 2013,^2015^; Kim et al., 2008; Malik et al., 2011; Wu et al., 2006). In brief, the Kalman filter is a recursive Bayesian estimation algorithm that infers the desired cursor state from the history of spike rates. Its “observation model” assumes that the baseline-subtracted spike rates *z* are linearly related to the intended movement direction *d* at each time point *t*:

(E1) $$z_{t}=Hd_{t}+q_{t}$$

where *H* is the matrix relating spiking activity to movement direction and the error term, *q*, is drawn from a normal distribution with zero mean and covariance matrix *Q*. Its “state model” assumes that the intended movement direction at any time evolves from the movement direction in the previous time point:

(E2) $$d_{t}=Ad_{t-1}+w_{t}$$

where *A* is the matrix relating movement directions at consecutive time points and the error term, *w*, is drawn from a normal distribution with zero mean and covariance matrix *W*.

#### Kalman filter calibration

The Kalman filter is calibrated by finding the parameters *H, Q, A, W* that maximize the log probability of jointly observing the set of intended movement directions *D* = {*d*_1_, *d*_2_, …, *d*_N_} and the set of spike rates *Z* = {*z*_1_, *z*_2_, …, *z*_N_}. Each channel’s mean spike rate was computed and subtracted from the ongoing rate separately for each block of data before the blocks were concatenated for Kalman filter calibration. Each feature was also normalized by its whole-block variance before block concatenation for decoder calibration.

To compute *D*, we assumed that the neural activity at each bin reflected the user’s intention to move the cursor directly toward the active target (Jarosiewicz et al., 2013), without making any assumptions about the intended cursor speed; thus, for calibration, we set *d_t_* to a unit vector pointing from the location of the cursor toward the location of the target. This causes the *H* matrix in the observation model to have units of Hz. In decoding, we converted the Kalman output back into velocity units using a gain factor tuned to each participant’s preference. We fixed *A* and *W* to provide a good trade-off between smoothness and responsiveness of cursor movement (Hochberg et al., 2012; Jarosiewicz et al., 2013). Thus, to calibrate the decoder, we only calculated the parameters *H* and *J* that maximized the joint distribution.

To initialize the decoder, the participant was asked to imagine moving a mouse on a tabletop plane with his right hand to move the cursor to targets presented one at a time in an 8-target radial center-out task. First, a 2-minute open-loop block was used to initialize the Kalman filter, and then three 3-minute closed-loop blocks with decreasing levels of error attenuation (0.8, 0.5 and 0.2) were used to refine it, as described in Jarosiewicz et al. (2013, 2015). As in these previous studies, this “standard” Kalman filter was updated after each center-out block under the assumption that the person was attempting to move the cursor directly toward each target. Only the first 3 s of each trajectory (after a 0.3 s delay) were used for calibration in an effort to reduce contamination of the neural signals by error correction. These intended direction vectors (all unit length) were regressed against the corresponding baseline-subtracted neural activity to obtain the tuning model and noise covariance matrix. After removing channels with intermittent noise, only the 40 features with the highest modulation with movement direction (Malik et al., 2015) were used in the decoder, chosen from all spike power features and the subset of threshold crossing features whose mean rates fell between 1 and 1,000 Hz.

#### Sequence game

The sequence game was inspired by and simplified from the electronic game “Simon” (https://en.wikipedia.org/wiki/Simon_(game)). Four radially displaced wedge-shaped targets were displayed on the screen (see Figure 1A; Videos S1 and S2). Each target was of a different color (yellow, red, dark blue and light blue), and each caused a different 200 ms-long musical note to be emitted from a speaker when “activated” (A4, B4, C5 and D5, respectively).

At the start of a trial, the neural cursor was displayed in the center of the screen as a sequence of 4 targets became brighter and then returned to baseline in series (0.5 s each for T9, and 0.75 s for T10, with no gap between targets; each target accompanied by its tone). Each target was used once in each sequence. The neural cursor then became active, and the participant was asked to move it through the same sequence he had just observed as quickly and accurately as possible. A target was acquired if the cursor dwelled on it for 0.3 s. If the correct target was acquired, the cursor was automatically re-centered, and the participant was able to continue to the next target. If an incorrect target was acquired, the trial ended and a cross was displayed at the center of the screen for 1.5 s while a low “error” tone was produced (duration = 0.2 s). At the end of a correctly repeated sequence, the time taken to acquire the full sequence was displayed in the center of screen for 1.5 s while a “celebratory” tone was produced (duration = 0.2 s). After a 0.5 s delay with a blank screen, the next trial began.

In each research session, 1 “repeated” and 11 different “control” sequences were used. The repeated sequence was always the same within a session but differed across sessions. The control sequences for a given session did not include any of the same target transitions as the repeated sequence, resulting in 11 possible 4-target control sequences.

In each block, 8 (or 16) sequences were presented. Each block consisted of 6 (or 12) presentations of the repeated sequences and 2 (or 4) pseudo-randomly interleaved presentations of the control sequences. The control sequences were presented in a pseudo-randomized order so that 1) each control was followed by at least 1 repetition of the repeated sequence; 2) all the control sequences were presented once before being presented again; and 3) a block did not start with a control sequence. In total in each research session, the repeated sequence was presented 66 times, and each of the 11 control sequences were repeated twice. In 4 of the research sessions, 1-2 of the same blocks were repeated because the participant expressed difficulty in controlling the neural cursor during the first presentation of that block.

#### Rest blocks and bias correction

In each rest block, the participant was instructed to close his eyes and was invited to take a nap. The duration of the rest blocks (20-30 minutes) were adjusted for each session according to the participant’s time constraints.

The rest blocks were performed just before (Rest1) and just after (Rest2) the sequence game blocks. This timeline permitted us to roughly match the amount of elapsed time between the Rest periods and the game period, so that any effects of neural signal nonstationarity didn’t differentially affect one of the rest periods more than the other.

However, because Rest1 took 20-30 minutes, neural signal nonstationarity could sometimes accumulate enough during the delay between decoder calibration and the start of the task to cause a velocity bias in cursor movement (Jarosiewicz et al., 2015) at the resumption of neural control. To reduce the detrimental effect of velocity bias on game performance, a short 4-min closed-loop center-out block was performed after the first rest period immediately before the first game block, with bias correction enabled (Jarosiewicz et al., 2015). Once bias was corrected, or if there was no noticeable bias during this test block, the closed-loop block was aborted and the sequence game was begun. Bias correction continued throughout the game blocks.

#### Template matching approach: sequenced single-target replay

The template matching method has been extensively used in animals (Euston et al., 2007; Johnson et al., 2010; Louie and Wilson, 2001; Ramanathan et al., 2015; Ribeiro et al., 2004; Tatsuno et al., 2006; Wilber et al., 2017). This method assesses the degree of similarity between spatiotemporal patterns of neural activity. Prior to creating neural activity templates, each channel’s features were adaptively z-scored over time with a 120 s time constant to reduce the effects of any nonstationarities in feature means and standard deviations (Jarosiewicz et al., 2016; Perge et al., 2013) that might otherwise spuriously affect correlations across time.

For the sequenced single-target replay analysis, the neural activity corresponding to each individual target was extracted from the repeated sequence trials in each session. Then, for each of the 4 targets, the mean duration of all trajectories to that target was obtained, and all of the neural activity sequences corresponding to that target were temporally resampled to that mean duration using MATLAB’s *resample* function. This allowed all neural activity patterns corresponding to that target’s trajectories to be averaged together, resulting in one neural activity template for each of the 4 targets. Thus, each average target’s template consisted of a TxF matrix, where T is the mean number of time bins across all trajectories to that target and F = 40 is the set of neural features.

Each single-target template was then compared to the adaptively z-scored firing patterns during the rest blocks using normalized 2D cross-correlation (Euston et al., 2007; see, e.g., Louie and Wilson, 2001; Tatsuno et al., 2006; Wilber et al., 2017). Local peaks in the correlation coefficient (CC) time series above the 95th centile threshold were identified, and candidate replay events were extracted from those peaks using non-maximum suppression, a machine learning algorithm that greedily selects the highest-scoring detections and suppress those that are already covered by (or overlap with) a higher nearby detection (see, e.g., Felzenszwalb et al., 2010; Rothe et al., 2015). In the present study, we enforced 0% overlap, which ensured that each replay event was only counted once-at its highest CC peak-even when there was more than one local maximum above the centile threshold within the duration of that template.

The 4 sets of single-target CC peaks were then interleaved into a single time series, with each peak labeled with its target ID (1,2,3, or 4). Each consecutive set of 4 targets (irrespective of their temporal spacing) was identified as either matching the order of targets in the repeated sequence (*hits*) or not (*misses*). The number of *hits* as a proportion of the total number of 4-target sequences (*hits* + *misses*) in each rest block were accumulated across all sessions for Rest1, and separately for Rest 2. Across sessions, the proportion of 4-target sequences corresponding to the repeated sequence was compared between Rest1 and Rest2 using a Chi-square proportion test.

#### Template matching approach: 4-target sequences

For the 4-target sequence template analysis, after the adaptive *Z* scoring described above, the activity pattern of *each* correctly played 4-target trial was used as a separate spatiotemporal template. This resulted in one TxF matrix for each sequence, where Tis the number of time bins in that trial (the time to play the sequences varied across trials; see Figure S1) and F = 40 is again the set of neural features that had been used in real-time decoding to control the movement of the cursor. Each individual template was compared to the rest blocks using normalized 2D cross-correlation (see Figure S2 for an example). Peaks in the resulting CC time series above a pre-specified centile threshold (candidate replay events) were selected using non-maximum suppression (as above) to ensure no overlap in template matches. For each template, the mean of the peak CCs was computed for Rest1 (m_1_) and Rest2 (m_2_), and a *Replay Index* (RI) was computed as the percent change from Rest1 to Rest2, RI = (m_2_ - m_1_) / m_1_. This process was repeated for all templates, resulting in one distribution of RIs for the repeated sequences, and one for the control sequences (see, e.g., Figure 2A). Within-sessions, the 2 distributions were then compared to each other using a two-sample 1-tailed t test to test whether the mean RI of the *repeated* sequence trials was significantly higher than the mean RI of the *control* sequence trials.

To test whether replay events were compressed or expanded in time (Csicsvari et al., 2007; Davidson et al., 2009; Diba and Buzsáki, 2007; Euston et al., 2007; Foster and Wilson, 2006; Ji and Wilson, 2007; Karlsson and Frank, 2009; Lansinket al., 2009; Lee and Wilson, 2002; Louie and Wilson, 2001; Wilber et al., 2017), we compressed and extended each trial template from 0.05 to 2.5x its original duration (time dilation factors = [0.05, 0.075, and 0.1 to 2.5 in increments of 0.1]) using MATLAB’s *resample* function and repeated the above analysis for all time dilation factors. We also verified that the results were not sensitive to the CC centile threshold we chose when identifying CC peaks by repeating the analysis using a sweep of different centile thresholds (75, 90, 95, 99 and 99.9). Results for each session are shown in Figure S3.

To test for replay across sessions, we first computed the mean RI of the *repeated* sequence trials and the mean RI of the *control* sequence trials in each session. We then tested whether the 10 *repeated*-trial mean RIs (1 for each session) were significantly higher than their respective *control*-trial mean RIs using a paired 1-tailed t test (see Figures 2B–2D and 3). This comparison was performed for each combination of timescale and CC centile threshold.

Multiple statistical comparisons were done (1) to ensure that the results were not sensitive to the choice of CC centile threshold and (2) to test for time compression or time dilation of replay. However, these comparisons were not fully independent of one another because (1) the set of peaks above any given CC centile threshold includes all of the peaks above higher centile threshold; and (2) neighboring time dilation factors result in nearly identical templates. Thus, a standard multiple-comparison correction does not readily apply to this analysis. Instead, to confirm that these results were not simply a spurious consequence of multiple comparisons, we also performed the identical number and type of t tests after swapping the control and repeated sequences. Under the null hypothesis, the number of significant outcomes resulting from these *swapped* t tests is expected to reflect the number of significant outcomes that would be obtained by chance.

#### Sleep versus wake delineation

Visual examination of the local field potentials (LFPs) collected from the implanted arrays during the rest blocks confirmed the occurrence of NREM1 sleep periods, marked by increases in power in the theta frequency band (4-7 Hz) (Gonzalez et al., 2018; Iber et al., 2007; Magnin et al., 2010). Thus, we used theta activity to separate putative waking from putative NREM1 periods during Rest1 and Rest2. Namely, the 30 ksps data acquired on each of the 96 channels on each array were converted to LFP signals in FieldTrip format (Oostenveld et al., 2011) and downsampled to 1kHz. A 2 Hz high-pass filter (zero-phase forward and reverse Butterworth filter, 6th order) was applied to remove slow deflection artifacts. For each array and block, LFP signals were averaged across all channels after excluding noisy (disconnected) or intermittently noisy channels, and the result was band-pass filtered for theta activity (4-7Hz) using a zero-phase forward and reverse finite impulse response (FIR) filter implemented in FieldTrip. A Hilbert transform was then applied to obtain the instantaneous envelope amplitude of the band-passed signal, and the instantaneous theta amplitude was averaged across the 2 arrays. Finally, the median theta amplitude was computed across all samples in each 20ms bin (aligning the theta amplitude bins with the stored firing rate bins). The mean (SD) theta amplitude across all bins in Rest1, in the sequence game, and in Rest 2 are displayed in Figure S5.

To define a theta threshold to delineate putative NREM1 periods, we used data acquired during the sequence game to give us an estimate of the baseline theta amplitude during waking. We defined the 80th percentile of theta amplitudes from the sequence game as the theta threshold; i.e., bins during rest periods exhibiting theta amplitude higher than this threshold were scored as “putative NREM1.” The number of bins used as “putative NREM1” and “putative waking” were equalized so that statistical tests could be compared between states without either set having a statistical power advantage (Table S2). To balance the number of bins, the same number of bins scored as “putative NREM1” were used for “putative waking” in each Rest period, but using the bins exhibiting the lowest theta amplitude. When more than 50% of bins in a given Rest period were identified as “putative NREM1,” the number of bins used for both was instead determined by the number of “putative waking” bins during that rest period.

### QUANTIFICATION AND STATISTICAL ANALYSIS

Two participants who were already enrolled in the BrainGate2 clinical trial participated in 5 research sessions for this study. As described in more detail in their respective sections above, statistical tests used were as follows. Behavioral comparisons were performed using 2-tailed Wilcoxon signed-rank tests to compare the speed and accuracy of acquiring the 4-target repeated versus control sequences. To test for sequenced single-target replay, a Chi-square proportion test was applied to compare the number of repeated sequence matches as a proportion of the total number of replayed 4-target sequences, accumulated across all sessions. To test for replay of the full 4-target sequences, a paired 1-tailed t test was applied across sessions (n = 10) to test whether the mean RI of the *repeated* sequence trials was significantly higher than the mean RI of the *control* sequence trial in each session. All statistical analyses were performed using MATLAB (https://www.mathworks.com/products/matlab.html). Statistical values, dispersion and precision measures (e.g., Mean, SEM) can be found in the figures, figure legends, and/or in the main text.

## Supplementary Material

1

2

3

## Figures and Tables

**Table:** KEY RESOURCES TABLE

REAGENT or RESOURCE	SOURCE	IDENTIFIER
Software and Algorithms		
Fieldtrip	(Oostenveld et al., 2011)	http://www.fieldtriptoolbox.org/
MATLAB	MathWorks	https://www.mathworks.com/products/matlab.html
Neuroport System	Blackrock Microsystems	https://www.blackrockmicro.com/neuroscience-research-products/neural-data-acquisition-systems/neuroport-daq-system/
SIMULINK	MathWorks	https://www.mathworks.com/products/simulink.html

## Highlights

- A brain-computer interface paradigm is used to test for memory replay in humans
- Spiking patterns that caused the learned movement sequences are replayed during rest
- Replay is stronger for learned than unlearned cursor movement sequences
- Replay occurs during both putative waking and NREM1 states
