# Supplementary material for: Replay of Learned Neural Firing Sequences during Rest in Human Motor Cortex
Source: Cell Rep. Author manuscript; Available in PMC 2020 Jul 6. (PMC7337233; doi:10.1016/j.celrep.2020.107581)
Supplement: 1 [file NIHMS1591481-supplement-1.pdf]

Cell Reports, Volume *31*

Supplemental Information

Replay of Learned Neural Firing Sequences during Rest in Human Motor Cortex

Jean-Baptiste Eichenlaub, Beata Jarosiewicz, Jad Saab, Brian Franco, Jessica Kelemen, Eric Halgren, Leigh R. Hochberg, and Sydney S. Cash

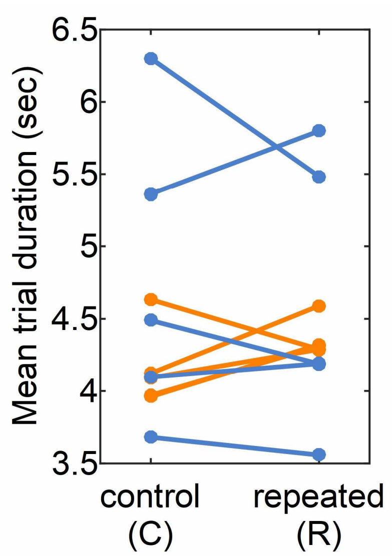

Figure S1. Related to Figure 1C. Average duration of the successfully-completed trials in each session. Mean time (in seconds) it took to finish the control (C) and repeated (R) sequence trials in each session (see **Figure 1C** for the % of C and R trials that were successfully completed). Each line represents one session from one participant (*orange* = T9 sessions; *cornflower blue* = T10 sessions). There was no difference in the amount of time it took to complete the R *vs.* C sequences (mean ± SEM, R: 4.50 ± 0.21 sec; C: 4.47 ± 0.25 sec).

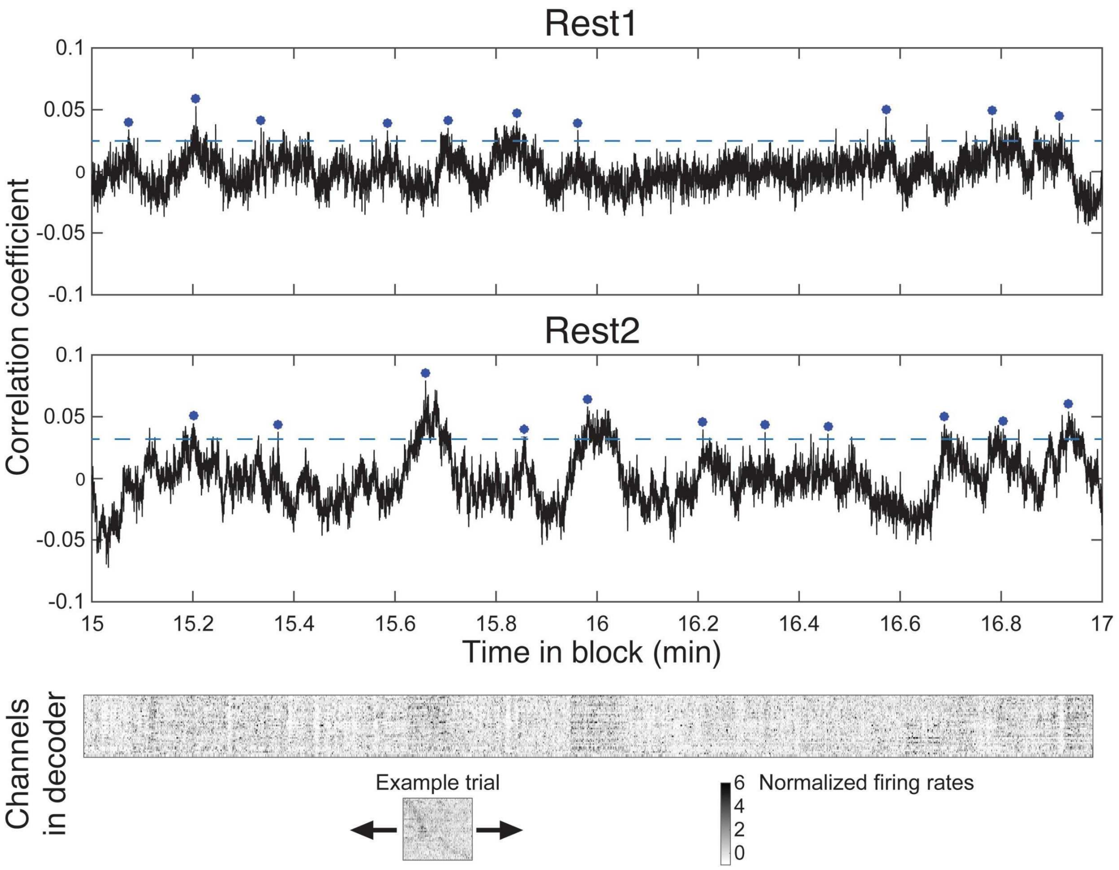

Figure S2. Related to Figures 2 and 3. Template matching methods. First, the normalized 2-D correlation coefficient (CC) was obtained between the template (the z-score normalized firing rates of the features used for decoding during one trial of the sequence task, or a time-dilated or time-compressed version thereof) and the firing rates of the same features across all timepoints of Rest1 and Rest2, sliding by 20 ms bins. An example of the firing rates during a repeated-sequence trial is shown at the bottom, and that of 2 minutes of a Rest2 period is shown just above it. The resulting CC time series for the same 2 minutes of Rest2 and for 2 minutes of Rest1 from the same session are shown in the top 2 panels. Then, the CC value at each local maximum above the specified CC centile threshold (blue dashed line) were obtained for each Rest period, and using non-max suppression, we ensured that only one peak (the highest local maximum) would be counted within a single template-length window (blue dots). The changes in mean CC peak values were then compared between Rest1 and Rest2 for all repeated sequence trials and all control trials, as described in *Template Matching Approach:4-target sequences* and in the main text.

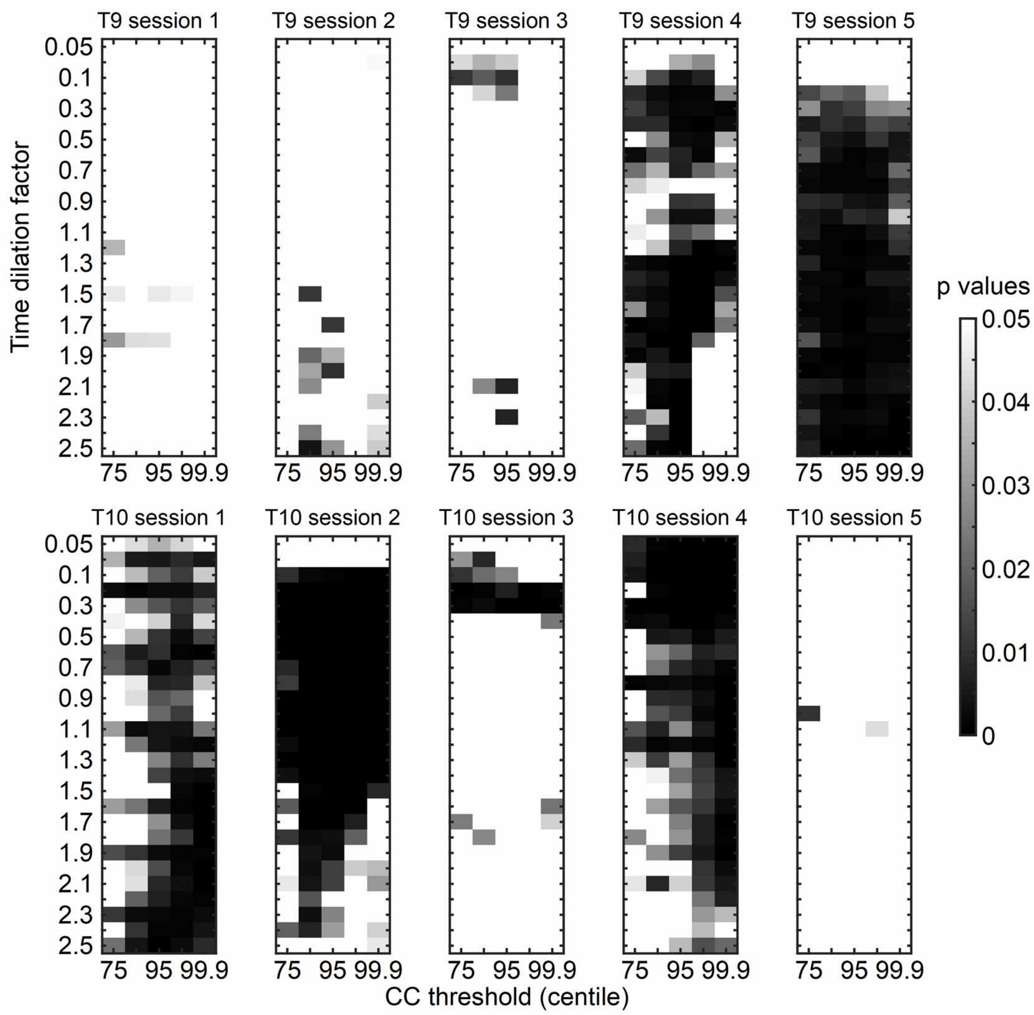

Figure S3. Related to Figure 2. Replay results for each session. The shading of each box represents the p value resulting from the two-sample 1-tailed t test (see **Figure 2A**) comparing the distribution of the *replay index* (% change in the peak CC values from Rest1 to Rest2) when using the repeated sequences as templates *vs.* the distribution resulting from using control sequences as templates (using the entire rest periods). Time dilation factors are along the y-axis, and CC centiles (75, 90, 95, 99, 99.9th centiles) above which peaks were taken are along the x-axis. All non-white boxes represent significant replay at $p < 0.05$ (see color scale). Note that, using the 95th centile CC threshold, significant replay was observed in 9 of the 10 sessions in at least one of the sampled timescales. Some of the variability across sessions in the magnitudes and timescales of replay could be attributable to variability in the amount of time spent in different physiological states. For example, T10's sessions 2 and 4 both had low theta power (consistent with more time spent awake) during the Rest periods (see **Figure S5**) than in the other sessions, and both of these sessions showed stronger replay at faster timescales. Other possible factors contributing to across-session variability might include the quality of memory encoding, the participant's engagement in the task, and other factors that could be examined in future studies.

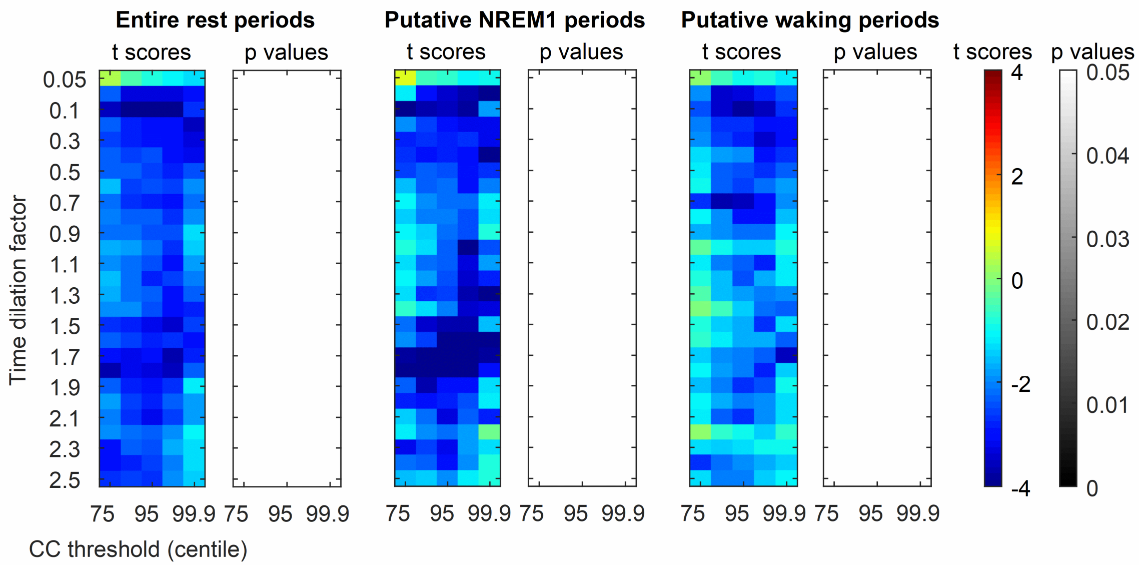

Figure S4. Related to Figures 2 and 3. Multiple comparisons do not account for the significant results we found. Here, *swapped* t tests were performed across sessions, testing whether RIs for the *control* sequences were significantly higher than RIs for the *repeated* sequences at any CC threshold or time dilation factor (paired 1-tailed t test, n = 10). This swapped t test analysis revealed no significant differences at any timescale or CC threshold, confirming that the significant replay of the repeated sequences relative to the control sequences was not simply a spurious consequence of multiple comparisons.

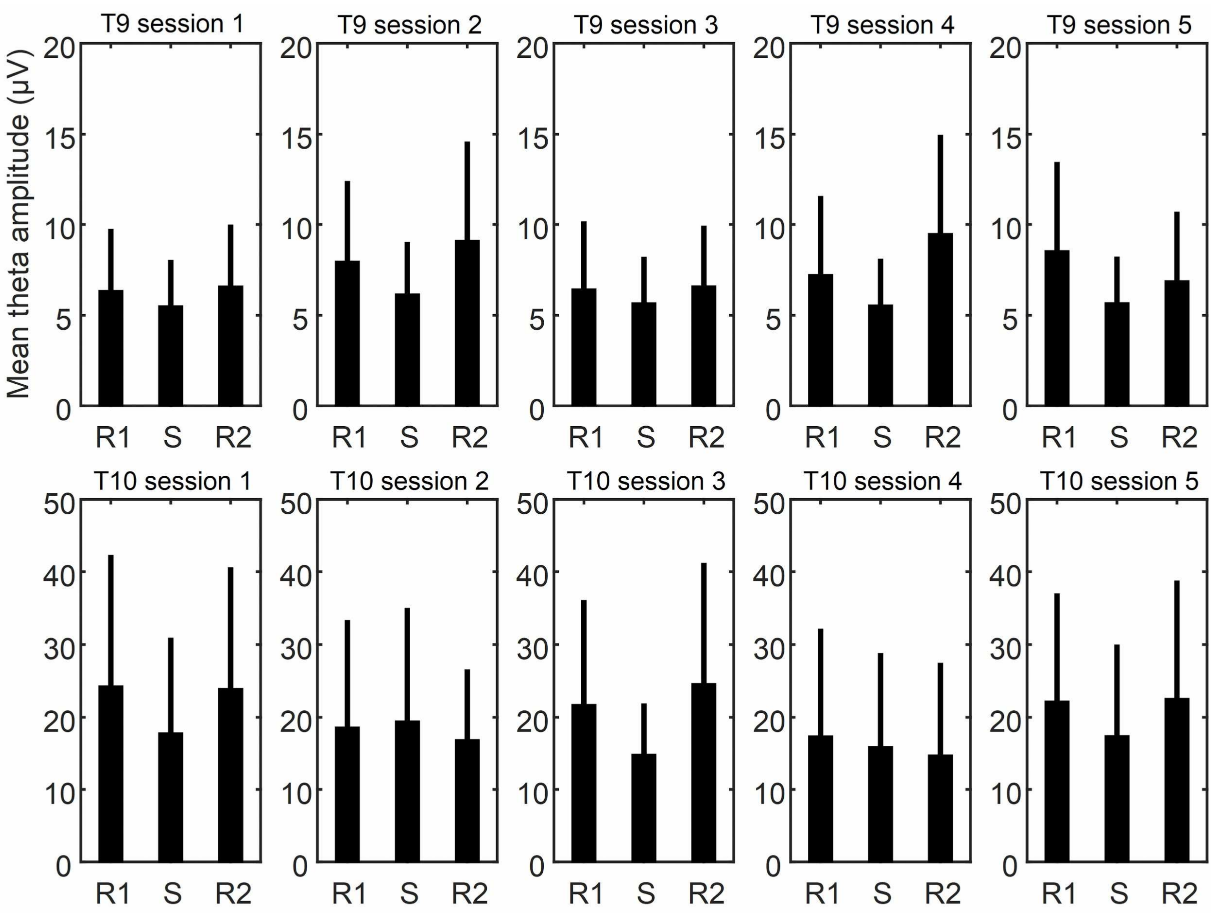

Figure S5. Related to Figure 3. Theta amplitude in each block of each session. For each session, the mean (error bars: SD) theta amplitude, computed as the envelope of the theta-filtered signal (4-7 Hz) using Hilbert transform, is shown for Rest1 (R1), the sequence game blocks (S), and Rest2 (R2).

Table S1. Related to Figure 1B. Summary of participants.

**ID**	**Gender**	**Age**	**Etiology**	**Electrode length (mm)**	***n* arrays**	**Time since implant**
**T9**	M	52	Amyotrophic lateral sclerosis (ALS)	1.5	2	11 months
**T10**	M	34	C4 spinal cord injury	1.5	2	3 months

Table S2. Related to Figure 3. Proportion of time spent in putative waking and NREM1 (and total amount of time, in minutes) in each rest period of each session. Time bins were identified as putative NREM1 if their theta amplitude exceeded a detection threshold set at the 80th percentile of the distribution of theta amplitudes during the task blocks. The putative waking bins were defined as those with the lowest theta amplitudes, matching the total number of bins in each rest period to those labeled putative NREM1 (see **Methods**). Because the % of time and total number of minutes spent in NREM 1 and putative waking were the same within a given rest period, this proportion (and time, in min) is given only once per rest period.

**Session**	**Rest1**	**Rest2**
T9 session 1	0.30 (6.04)	0.34 (7.31)
T9 session 2	0.38 (9.60)	0.46 (11.59)
T9 session 3	0.28 (5.61)	0.32 (9.54)
T9 session 4	0.39 (7.71)	0.41 (12.17)
T9 session 5	0.50 (12.48)	0.34 (10.25)
T10 session 1	0.40 (9.99)	0.39 (9.80)
T10 session 2	0.22 (5.52)	0.15 (3.73)
T10 session 3	0.44 (11.09)	0.49 (12.29)
T10 session 4	0.24 (6.09)	0.14 (3.63)
T10 session 5	0.39 (9.83)	0.39 (9.80)
**Mean**	**0.35 (8.40)**	**0.34 (9.01)**
**SD**	**0.09 (2.52)**	**0.12 (3.17)**
